# Polio eradication: next steps and future challenges

**DOI:** 10.2807/1560-7917.ES.2018.23.47.1800625

**Published:** 2018-11-22

**Authors:** Maria Zambon, Javier Martin

**Affiliations:** 1 National infection Service, Public Health England, London, United Kingdom; 2 National Institute for Biological Standards and Controls (NIBSC), Potters Bar, United Kingdom

**Keywords:** polio, poliomyelitis, polio vaccine, vaccination, bOPV, tOPV, polio eradication, polio biocontainment, Acute Flaccid Paralysis (AFP), vaccine derived poliovirus (VDPV), inactivated polio vaccine (IPV)

In 1988, the World Health Assembly resolved to eradicate polio. At that time, polio was endemic in 125 countries and paralysed around 1,000 children per day. Since then, polio cases due to infection with wild poliovirus (WPV) have decreased by more than 99.9% from over 350,000 cases a year to 37 cases in 2016 and 22 in 2017 [1]. Of the three WPV serotypes, 1, 2 and 3, WPV2 has not been detected since 1999 and was declared eradicated in September 2015. This allowed a global switch from live trivalent oral polio vaccine (tOPV) to live bivalent oral polio vaccine (bOPV), eliminating the need for live type 2 poliovirus vaccine strains. At the same time, this switch has created a need for universal use of inactivated polio vaccine (IPV) to ensure immune protection against type 2 poliovirus. Vigorously applied disease control programmes have clearly made huge contributions to the goal of eradication of poliomyelitis, and we are tantalisingly close to the complete elimination.

Clinical and virological surveillance using the acute flaccid paralysis (AFP) case definition, in tandem with comprehensive vaccination programmes using OPV have been extremely successful in high disease burden countries. WPV3 was last detected in November 2012 in Nigeria, and since this time WPV1 has been the sole circulating WPV type globally. WPV transmission has persisted in only two countries: Afghanistan and Pakistan, although in August 2016 it was also detected in Nigeria [1]. As we begin to see the light at the end of the tunnel, a relentless focus on achieving complete vaccination coverage in the areas still using OPV, together with a global commitment to universal IPV coverage and diverse approaches to surveillance, are needed to achieve the final target.

## Endgame strategy

The Polio Eradication and Endgame Strategic Plan (2013–18) set the goal of a polio-free world by the end of 2018 [2]; this was recently extended until at least 2021 [3]. Achieving this challenging goal requires: (i) completion of WPV eradication to eliminate the risk of WPV transmission; (ii) cessation of the use of oral polio vaccine (OPV) after eradication completion to eliminate the risks of chronic immunodeficiency-associated vaccine-derived poliovirus (iVDPV) cases and circulating vaccine-derived poliovirus (cVDPV) causing outbreaks due to person-to-person transmission in areas with poor vaccination coverage [4,5]; and (iii) implementation of poliovirus safe-handling and containment measures following the World Health Organization (WHO) Global Action Plan (GAPIII) to minimise poliovirus facility-associated risk after type-specific eradication of wild polioviruses and sequential cessation of oral polio vaccine use to minimise the risks of reintroduction of virus into the polio-free community [6]. The last pockets of WPV circulation are in Afghanistan and Pakistan, where the physical challenges of landscape and isolated communities are compounded by a challenging socio-political environment that affects the timely delivery of vaccines [7]. The commitment of local health authorities is slowly but gradually leading to improvements in vaccine coverage [8]. Delivery of vaccine programmes has never been more essential.

## Clinical surveillance

The assessment of polio elimination status in a country is based upon demonstration of routinely high uptake of vaccine in children and evidence of strong polio surveillance. One of the hallmarks of the smallpox elimination campaign in its final stages in the 1970s was relentless tracking and detailed investigations of possible cases, however difficult the circumstance or how improbable the clinical case. Smallpox had a distinct clinical presentation making it easier to recognise compared with polio, where the key indicator clinical syndrome for the elimination is acute flaccid paralysis (AFP). This occurs in less than 5% of poliovirus-infected individuals, and is also the result of poorly understood aetiologies such as Guillain-Barré syndrome. Currently, many countries struggle to undertake AFP surveillance. Polio-associated AFP is a rare disease overall, and its declining incidence and lack of perceived importance has led to difficulties in use and verification of individual cases [3]. As discussed in this week’s *Eurosurveillance* report of the Spanish experience of AFP surveillance over the past 20 years, in at least a third to two thirds of cases the supporting virological investigations may also be less than optimum [9]. The findings emphasise that the overall sensitivity of passive AFP case finding, as a tool for detection of polio circulation in the era of eradication, is insufficient and needs to be supplemented. Awareness raising within the clinical and paediatric communities, of the importance of timely notification of possible AFP cases and detailed and disciplined investigation, is necessary to overcome the presumption that polio has indeed disappeared and to dispel the notion that case follow-up effort is no longer required. Inadequate sampling for virological investigation is a risk to clinical surveillance programmes. The widest range of samples, including respiratory and faecal samples, should be analysed from each possible AFP case as part of a carefully coordinated approach to thoroughly investigate causality. Virological analysis of body fluids from AFP cases and other polio-compatible neurological illnesses is a crucial tool in verifying the lack of circulating polioviruses. The gradual shift in clinical practice towards investigating possible infectious episodes of acute onset neurological illness using PCR with cerebrospinal fluid (CSF) as the specimen of choice will further reduce the sensitivity of AFP surveillance. Using well-timed faecal samples for virological investigations, as recommended in the WHO Polio laboratory manual [10], provides a much higher chance to detect poliovirus infection.

Correct classification of poliovirus isolates as vaccine-like strains, VDPVs and WPVs, through a competent laboratory network, is essential to support rapid and thorough outbreak investigations and to help orchestrate the best public health response. Tried and tested classical laboratory methodologies involving selective cell culture systems for virus isolation followed by molecular typing methods are very sensitive for the detection of WPV and VDPV strains but, as yet, fully validated direct detection methods for poliovirus identification in stool extracts are not available. As mentioned above, WPV1 is still circulating in areas of Pakistan and Afghanistan. In addition, cVDPVs are still causing outbreaks in various countries of Africa (Democratic Republic of Congo, Nigeria, Niger and Somalia) and in Papua New Guinea [1]. Until poliovirus transmission is interrupted, all countries remain at risk of importation of PV. Especially vulnerable are countries with inadequate public health and immunisation services and travel or trade links to endemic countries. With the eradication of WPV2, it is particularly important to focus on rapid response to cVDPV2 outbreaks and to ensure there is heightened biosecurity for WPV2 held in laboratory facilities.

## Environmental surveillance

Work has been undertaken to develop alternative surveillance approaches to verify that there are no circulating polioviruses. This includes virus isolation and detailed molecular characterisation of enteroviruses recovered from severe, neurological illness cases [11], and environmental surveillance (ES) of waste water and sewage systems [12]. Unlike clinical (AFP) surveillance, ES can monitor large populations using smaller numbers of samples and detect the introduction of poliovirus even before the appearance of AFP cases, and can be a sensitive tool to pick up circulation of WPV or VDPV. The silent transmission of type 1 WPV in 2013 in Israel has demonstrated the value of active ES that can detect poliovirus excretion in the population in the absence of polio-related AFP cases [13]. While the application of advanced molecular genomics tools for the analysis of complex environmental samples will add sophistication and improve current analytical approaches [14], there are substantial logistical and financial constraints in implementing ES at scale in densely populated countries. Such approaches require detailed consideration of sampling strategies and cross sectoral collaborations involving parties outside the primary health system. Implementation of ES programmes in a rational way will support strengthened clinical surveillance programmes, though much more work is needed to refine sampling strategies and develop standardised sampling and laboratory analytical methods.

## Biocontainment

As the focus of the global eradication programme shifts away from tracking the transmission of WPV towards detailed oversight of poliovirus-related laboratory and manufacturing activities at national level, there is now a need to give greater emphasis to the regulation of biocontainment. The report in 2017 of two accidental exposures to WPV-2 in a Dutch manufacturing plant [15] was a wake-up call on what might be the consequences of breaches in biocontainment or gaps in scenario planning and emphasised the need for particular vigilance in these facilities worldwide. The incident also highlighted the necessity of having detailed public-health risk-management plans ready for uncontrolled and unintended release events arising in laboratory environments. This aspect has been addressed in Europe by detailed simulation exercises led by WHO in autumn 2018 to test national plans and preparedness for such events. In addition, recent reports of tOPV use in India that led to the exposure of children to live type 2 vaccine strain after the tOPV to bOPV switch [16,17] showed that stringent regulatory oversight of vaccine manufacturing facilities is absolutely required. The risks associated with poliovirus vaccine production, including IPV, are now magnified in the absence of circulating viruses. These include the inadvertent release of large volumes of biological materials, potentially containing high titres of live poliovirus to the sewage system, as part of the IPV vaccine production process. When this happened on a previous occasion, it required a major public health response and complex investigations to assess the impact of possible large-scale environmental contamination [18].

## Polio essential facilities

Under the WHO GAPIII [6], it is envisaged that the number of facilities holding and working with WPV will decrease markedly. Countries will be required to establish a national authority for containment (NAC) by the end of 2018, to register as polio essential facilities (PEFs) organisations handling poliovirus within their borders and to provide oversight of their activities. NACs will scrutinise critical testing functions that require the use of live poliovirus much more stringently. These will include vaccine manufacture and laboratory activities, such as measuring population immunity and immunogenicity testing and preparation of standards and controls for diagnostic assays. The risks associated with poliovirus vaccine production, including IPV, are now magnified in the absence of circulating viruses, as manufacturing facilities will be the single largest source of live poliovirus. Implementation deadlines for registration of PEFs and enhanced regulation of biocontainment appear challenging, but the necessity of increased regulatory oversight at this stage of the end game, while recognising the need to ensure global IPV availability, is uncontroversial, even without the examples above.

During the coming 12 to 18 months, the work of National Accreditation Committees should generate a greater understanding of the distinction between the activities that require a PEF and how to classify and handle poliovirus infectious and potentially infectious materials (IMs and PIMs) [19]. The committees will support GAPIII processes and serve to strengthen biological risk management in facilities delivering critical functions. Global research priorities for a virus that is being eradicated link directly to risk reduction associated with polio vaccine manufacture, where there is no alternative to the use of live virus. Some of the biocontainment risk associated with continuing use of OPV could be addressed through the use of safer, genetically stabilised, live-attenuated vaccine strains. Similarly, the use of hyperattenuated poliovirus strains such as (S19) for vaccine production, quality assurance programmes and immunogenicity testing would improve risk management during the IPV vaccine manufacturing process [20]. These and other related research activities will require the use of live poliovirus strains as gold standards for some years to come, with the expectation that ultimately the fruits of the labour will reduce the global biorisk of polio vaccine manufacture.

We have come a long way since the March of Dimes was launched almost 100 years ago in the United States as the first popular societal movement against polio. Today’s achievements in polio eradication arise from the combined and collaborative efforts of many different international health agencies, private foundations and governments and individuals being vaccinated, each playing their part. Taking a backwards look is a reminder of the scale of the challenge and the immense human effort that has been required to get us to where we are now. With the end in sight, the final push towards complete polio eradication, to overcome the remaining challenges, requires a last heave and concerted effort from many different sectors to finally eliminate the scourge of polio-associated paralysis.
